# Weight-gain independent effect of mirtazapine on fasting plasma lipids in healthy men

**DOI:** 10.1007/s00210-023-02448-y

**Published:** 2023-03-09

**Authors:** Katharina Lechner, Sarah Heel, Manfred Uhr, Tatjana Dose, Florian Holsboer, Susanne Lucae, Ludwig Schaaf, Stephany Fulda, Stefan Kloiber, Johannes M. Hennings

**Affiliations:** 1 Max Planck Institute of Psychiatry, Munich, Germany; 2 Department of Cardiology, German Heart Centre Munich, Technical University Munich, Munich, Germany; 3 DZHK (German Centre for Cardiovascular Research), Partner Site Munich, Munich Heart Alliance, Munich, Germany; 4 Clinic for Neuroendocrinology and Andrology, Max Planck Institute of Psychiatry, Munich, Germany; 5 Neurocenter of Southern Switzerland, EOC, Lugano, Switzerland; 6 Centre for Addiction and Mental Health, Campbell Family Mental Health Research Institute, Toronto, ON Canada; 7 Department of Psychiatry, Institute of Medical Science, Department of Pharmacology and Toxicology, University of Toronto, Toronto, ON Canada; 8 Department of Dialectical Behavioral Therapy, Kbo-Isar-Amper-Klinikum München-Ost, Vockestraße 72 85540 Haar/Munich, Germany

**Keywords:** Mirtazapine, Atherogenic dyslipidemia, TG/HDL-C ratio, Body weight, Cardiovascular risk

## Abstract

Treatment with mirtazapine, a widely prescribed antidepressant, has been linked to weight gain and dyslipidemia. Whether dyslipidemia occurs secondary to increased appetite due to antidepressant treatment, or due to direct pharmacological effects of mirtazapine is unknown. The aim of this analysis is to complement our previously published results of the effect of mirtazapine on metabolism and energy substrate partitioning from a proof-of-concept, open-label clinical study (ClinicalTrials.gov NCT00878540) in 12 healthy males (20–25 years). We report the effect of a seven-day administration of mirtazapine 30 mg per day on weight and lipid metabolism in healthy men under highly standardized conditions with respect to diet, physical activity and day-night-rhythm and under continuous clinical observation. After a 7-day administration of mirtazapine 30 mg, we observed a statistically significant increase in triglyceride levels (mean change + 4.4 mg/dl; 95% CI [– 11.4; 2.6]; *p* = 0.044) as well as TG/HDL-C ratio (mean change + 0.2; 95% CI [– 0.4; 0.1]; *p* = 0.019) and a decrease in HDL-cholesterol (mean change – 4.3 mg/dl; 95% CI [2.1; 6.5]; *p* = 0.004), LDL-cholesterol (mean change – 8.7 mg/dl; 95% CI [3.8; 13.5]; *p* = 0.008), total cholesterol (mean change – 12.3 mg/dl; 95% CI [5.4; 19.1]; *p* = 0.005), and non-HDL-C (mean change – 8.0 mg/dl; 95% CI [1.9; 14.0]; *p* = 0.023). Notably, weight (mean change – 0.6 kg; 95% CI [0.4; 0.8]; *p* = 0.002) and BMI (mean change – 0.2; 95% CI [0.1; 0.2]; *p* = 0.002) significantly decreased. No change in waist circumference (mean change – 0.4 cm; 95% CI [– 2.1; 2.9]; *p* = 0.838) or waist-to-hip-ratio (mean change 0.0; 95% CI [– 0.0; 0.0]; *p* = 0.814) was observed. This is the first study showing unfavorable changes in lipid metabolism under mirtazapine in healthy individuals despite highly standardized conditions including dietary restriction, and despite the observation of a decrease of weight. Our findings support the hypothesis that mirtazapine has direct pharmacological effects on lipid metabolism. ClinicalTrials.gov: NCT00878540.

## Introduction

Mirtazapine is a noradrenergic and specific serotonergic antidepressant (NaSSA) that is widely used in the treatment of major depressive disorder. (Croom et al. 2009) Its antidepressant effect is mainly attributed to enhanced noradrenergic and serotonergic (5-HT_1_-mediated) activity (Boer et al. 1994). Mirtazapine acts as an antagonist at central α_2_-adrenergic auto- and heteroreceptors and at postsynaptic 5-HT_2_- and 5-HT_3_- receptors (Puzantian 1998). It has no significant influence on dopamine metabolism but acts as a strong antagonist at histamine H_1_-receptors. As histamine plays a central role in appetite regulation, H_1_-receptor antagonism may account for adverse effects during treatment with mirtazapine such as weight gain (Croom et al. 2009).

Patients treated with mirtazapine frequently report adverse drug effects, most frequently weight gain and sedation (Gartlehner et al. 2008; Himmerich et al. 2006; Kraus et al. 2002; Nicholas et al. 2003; Watanabe et al. 2010; Zimmermann et al. 2003). Weight gain may, beyond limiting adherence, result in metabolic dysregulation, which aligns with the observation that pharmacotherapy with mirtazapine is associated with dyslipidemia (Nicholas et al. 2003; Hennings et al. 2019; Roose 2003). Regarding dyslipidemia, treatment with mirtazapine is most consistently associated with a rise in triglycerides (Nicholas et al. 2003). Hypertriglyceridemia is commonly observed in the context of a lipoprotein pattern referred to as atherogenic dyslipidemia, which encompasses a constellation of lipoprotein abnormalities, including high serum triglycerides and reduced large HDL particles (and/or low HDL-C), as well as an atherogenic lipoprotein phenotype, including a predominance of small, cholesterol-depleted LDL particles, and an accumulation of triglyceride-rich remnant lipoproteins. Atherogenic dyslipidemia is the central lipoprotein phenotype associated with truncal adiposity, elevated intrahepatic triglyceride pool (i.e., non-alcoholic fatty liver disease), insulin resistance, and type 2 diabetes mellitus (Lechner et al. 2020a, 2021). It both clinically and pathologically closely tracks with atherosclerotic cardiovascular disease (ASCVD) risk (Lechner et al. 2020a). This raises possible concerns about the cardiovascular safety of mirtazapine. The effects of mirtazapine on total cholesterol (TC), LDL-cholesterol (LDL-C), and HDL-cholesterol (HDL-C) are heterogeneous (Nicholas et al. 2003; McIntyre et al. 2010, 2006).

While previous data on the effect of mirtazapine on glucose tolerance in psychiatric patients have been conflicting (Fisfalen and Hsiung 2003; Hennings et al. 2019; Hennings et al. 2012), our recent analysis in healthy men revealed a shift in energy substrate partitioning towards carbohydrate substrate preference assessed by indirect calorimetry under highly standardized conditions including dietary restriction (Hennings et al. 2019). Further, insulin and C-peptide release increased in response to a standardized meal suggesting weight-gain independent, direct pharmacological (i.e., not secondary to increased appetite and food intake but direct effects on energy substrate metabolism) effects of mirtazapine (Hennings et al. 2019).

Dyslipidemia and hypertriglyceridemia in particular are highly responsive to variables such as diet, changes in body weight, insulin sensitivity, and sympathetic tone (Kopf et al. 2004). Changes in lipid metabolism during treatment with mirtazapine have been accompanied by an increase in body weight in prior studies—often interpreted as a result of increased appetite and changed eating behavior induced by the drug (i.e., non-direct pharmacological effects) (Nicholas et al. 2003; Zimmermann et al. 2003). Nevertheless, possible direct effects of mirtazapine on hepatic lipid metabolism independent of weight-gain have not yet been investigated under standardized conditions and biological mechanisms of mirtazapine-associated dyslipidemia remain unclear.

Thus, we investigated the effect of mirtazapine 30 mg daily administered over 7 days on weight and fasting plasma lipid profiles in healthy individuals in a highly standardized setting with regard to nutrition, exercise and sleep–wake-rhythm. This analysis complements our previously published findings of the effects of mirtazapine on glucose metabolism and energy substrate partitioning (Hennings et al. 2019).

## Methods

### Study design

**Fig. 1:**
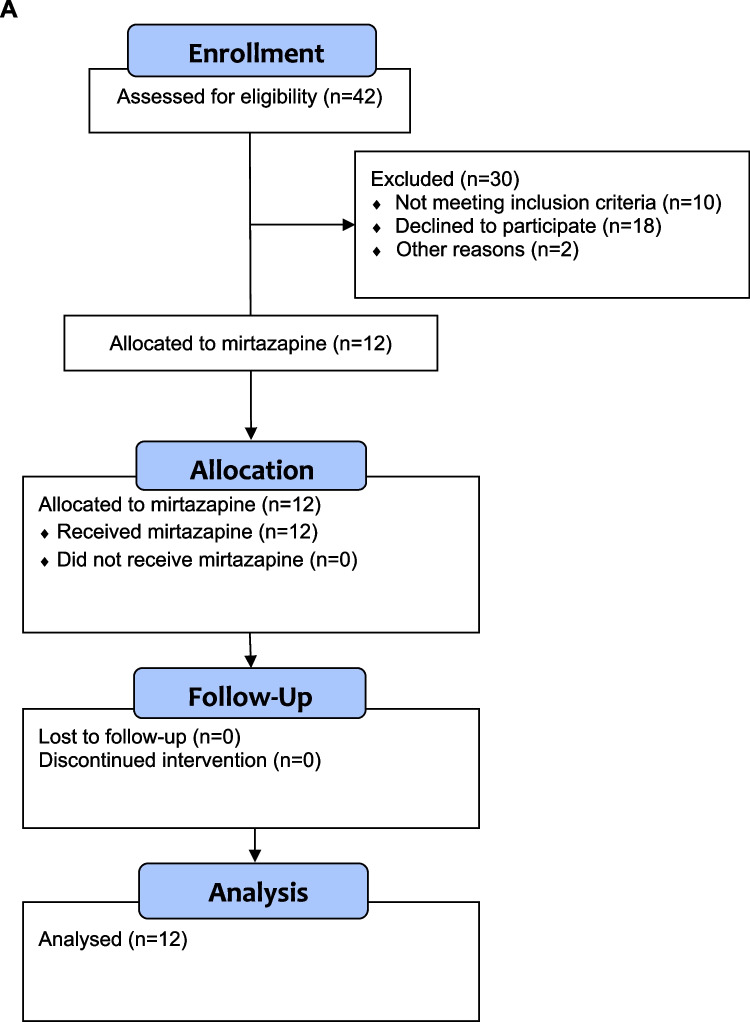

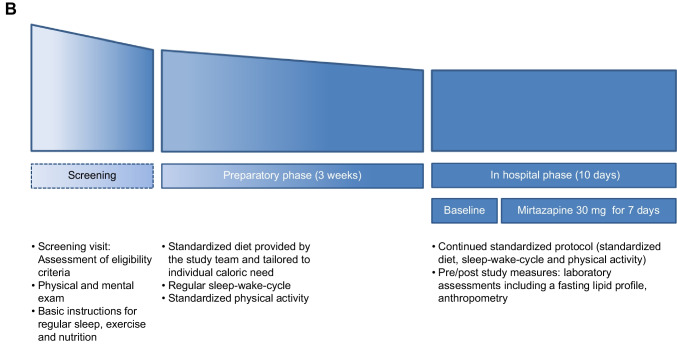
**A** Study flow chart. **B** Study protocol. The narrowing bars in the upper part of (**B**) depict the increasing extent of monitoring with regard to sleep, physical activity, and caloric intake throughout the study period: instructions for regular sleep, exercise, and nutrition within the screening phase; assessment of hunger/satiety, adaptation of caloric need, sleep diaries, and activity protocols within the preparatory phase; and, finally, continuous 24 h/day monitoring and the continuation of standardized diet, sleep/wake cycle, and physical activity during the inward phase

The study followed a prospective, non-randomized design (ClinicalTrials.gov NCT00878540) involving a homogenous and comprehensively characterized cohort of 12 healthy male volunteers of European ancestry aged 20 to 25 years to study the effect of a 7-day exposure to mirtazapine 30 mg daily on lipid metabolism in a highly standardized setting. Volunteers were recruited via postings at university bulletin boards and through ads on the website of the Max-Planck-Institute. Prior to the initiation of any study related procedures, all study participants gave written informed consent. Standardization with respect to sleep, physical activity, and caloric intake was initiated at the beginning of the preparatory phase, which started 3 weeks prior to the 10-day hospital phase that included the 7-day mirtazapine treatment period. Participants were instructed to initiate standardization and were provided standardized meals for the entire time period from the beginning of the 3-week preparatory phase throughout the study period which included the 10-day hospital phase, though level of monitoring increased as participants were continuously observed during the hospital phase. The study design has been published (Hennings et al. 2019) and is shown in Fig. 1A and B.

While in our previous analysis, two participants needed to be excluded due to protocol violation related to the calorimetry, the current analysis was not affected hereby, and we report on the full data set of 12 individuals. All study participants were allocated to a standardized 3-week ambulatory accommodation phase. Caloric need was individually determined based on age, weight, height, and basal metabolic rate as assessed by indirect calorimetry by a registered dietician according to the recommendations of the German Nutrition Society (www.dge.de). Of note, participants were asked to regularly engage in a moderate intensity physical activity regimen prior to the start of the preparatory phase to adjust the basal metabolic rate (as assessed by indirect calorimetry prior to the preparatory phase) and caloric intake to the subsequent study phases. Caloric intake was then kept constant throughout the study period, as was physical activity and sleep–wake rhythm. Besides water, any additional drinks were prohibited, as was any additional food. During the 10-day experimental phase, participants were under continuous clinical observation, maintained standardized diet, sleep–wake cycle, and physical activity regimen (2 h of light walking per day, no exercise training). After a 3-day adaptation period to the hospital setting, 30 mg mirtazapine was given (Remergil SolTab, Organon) at 10 pm daily for 7 days. Compliance was ensured by the presence of qualified study staff during administration of the medication and by measurement of mirtazapine plasma levels. Time spent in bed was standardized (11:00 p.m. to 07:00 a.m.) during the entire study period (preparatory phase and experimental phase).

### Study population

Participants were eligible for the study if they were healthy males of European ancestry between 18 and 25 years of age with normal BMI (18.5–25 kg/m^2^). Further eligibility criteria included being completely healthy and having a regular sleep wake rhythm. Participants were excluded if they had a history of or first-degree relatives with psychiatric or metabolic disorders. Individuals with current and/or a history of mental disorders as assessed by the Diagnostic and Statistical Manual of Mental Disorders (DSM) IV axis I were excluded. Further exclusion criteria were significant abnormalities in laboratory or physical examinations, substance use (including any cigarette smoking within the last 6 months or abuse of alcohol or other illicit drugs), medication use during the past 6 months (except infrequent use of medication for pain (excluding opioids) until 2 weeks prior to the start of the study), high performance sport, shift work, or time-zone travel in the previous 12 months. The full list of eligibility criteria may be obtained at ClinicalTrials.gov NCT00878540.

Current health status and past history were assessed by a detailed medical history, physical examination, routine blood draw (including complete blood count, liver, renal, and thyroid function and lipid profile) and urinalysis, electrocardiogram, electroencephalogram, and cranial magnetic resonance imaging (cMRI). The presence of a current and/or former psychiatric disorder according to DSM IV axis I was assessed with the modified version of the Munich-Composite International Diagnostic Interview (DIA-X/M-CIDI) (Wittchen et al. 1998). All study participants had a regular circadian rhythm without evidence of sleep disorders or daytime sleepiness as assessed with interviews, questionnaires, and sleep diaries (this has been comprehensively described previously) (Fulda et al. 2013). Participants were recruited at the Max Planck Institute of Psychiatry, Munich, Germany, between December 2008 and April 2010.

### Assessments

#### Laboratory measurements

For lipid analysis, venous blood was drawn at 08.30 am after 12 h of fasting from an antecubital vein using standard venipuncture techniques at baseline (i.e., morning of the first drug day) and after administration of mirtazapine for 7 days (i.e., morning after drug day 7). Blood samples were immediately processed. A standard lipid panel (TC, HDL-C, LDL-C, and triglycerides) was done using an enzymatic assay (Roche Hitachi 912). Non-HDL-C and triglycerides to HDL-C ratio were calculated as follows: non-HDL-C = TC-HDL-C and triglycerides to HDL-C ratio = TG/HDL-C.

### Other variables

Weight was measured in kilograms on a calibrated scale at 07.30 am after urination. BMI was calculated: BMI = kg/(m^2^). Waist and hip circumference were measured in cm with waist to hip ratio being the quotient of these measures.

### Statistical analysis

Data were analyzed using the non-parametric paired sample two-sided Wilcoxon signed rank test. Results are reported as mean ± SD or mean changes with 95% confidence intervals (CI) analyzed by paired *t*-tests. A significance level of α = 5% was used for all tests. As all tests were hypothesis generating without confirmatory interpretation, no correction was applied to counteract the problem of multiple comparisons. All statistical analyses were performed using IBM SPSS Statistics for Windows, version 25 and 28 (IBM Corp., Armonk, NY, USA).

### Ethics

The study was approved by the competent authorities and the Ethics Committee of the Medical Faculty at the Ludwig Maximilians University, Munich, Germany [ethics approval on 07–18-2008 (EudraCT: 2008–002,704-26)]. Written informed consent was obtained from all subjects. The study was carried out in accordance with the version of the Declaration of Helsinki that was available at the time of conceptualization of the study.

## Results

Baseline characteristics and weight, body composition, and lipid profiles following the intervention are shown in Table 1.

**Table 1:** Baseline characteristics and weight, waist circumference and lipid profiles at baseline and following exposure to mirtazapine^A^

	Baseline		Post-intervention		Z	P^B^	Mean change	95% CI
	M	SD	M	SD				
Age (years)	22.3	1.5						
Height (cm)	180.7	6.9						
Weight (kg)	74.4	5.9	73.8	5.8	– 3.1	**0.002**	– 0.6	0.4; 0.8
Body-mass-index (kg/m^2^)	22.8	1.6	22.6	1.6	– 3.1	**0.002**	– 0.2	0.1; 0.2
Waist circumference (cm)	78.9	4.2	78.5	2.9	– 0.2	0.838	– 0.4	– 2.1; 2.9
Waist/hip ratio	0.9	0.0	0.9	0.0	– 0.2	0.814	0.0	– 0.0; 0.0
Total cholesterol (mg/dl)	146.6	25.1	134.3	21.1	– 2.8	**0.005**	– 12.3	5.4; 19.1
Triglycerides (mg/dl)	64.0	19.3	68.4	17.4	– 2.0	**0.044**	4.4	– 11.4; 2.6
HDL-C (mg/dl)	47.5	5.6	43.3	5.5	– 2.9	**0.004**	– 4.3	2.1; 6.5
LDL-C (mg/dl)	85.8	20.8	77.2	17.2	– 2.7	**0.008**	– 8.7	3.8; 13.5
Non-HDL-C (mg/dl)	99.0	23.4	91.1	19.4	– 2.3	**0.023**	– 8.0	1.9; 14.0
TG/HDL-C	1.4	0.4	1.6	0.5	– 2.4	**0.019**	0.2	– 0.4; 0.1

^A^Data (*n* = 12) are mean values (M) and standard deviations (SD).

^B^Two-sided significance p < 0.05 is in bold (two-sided Wilcoxon signed rank test).

### Lipid metabolism

**Fig. 2:**
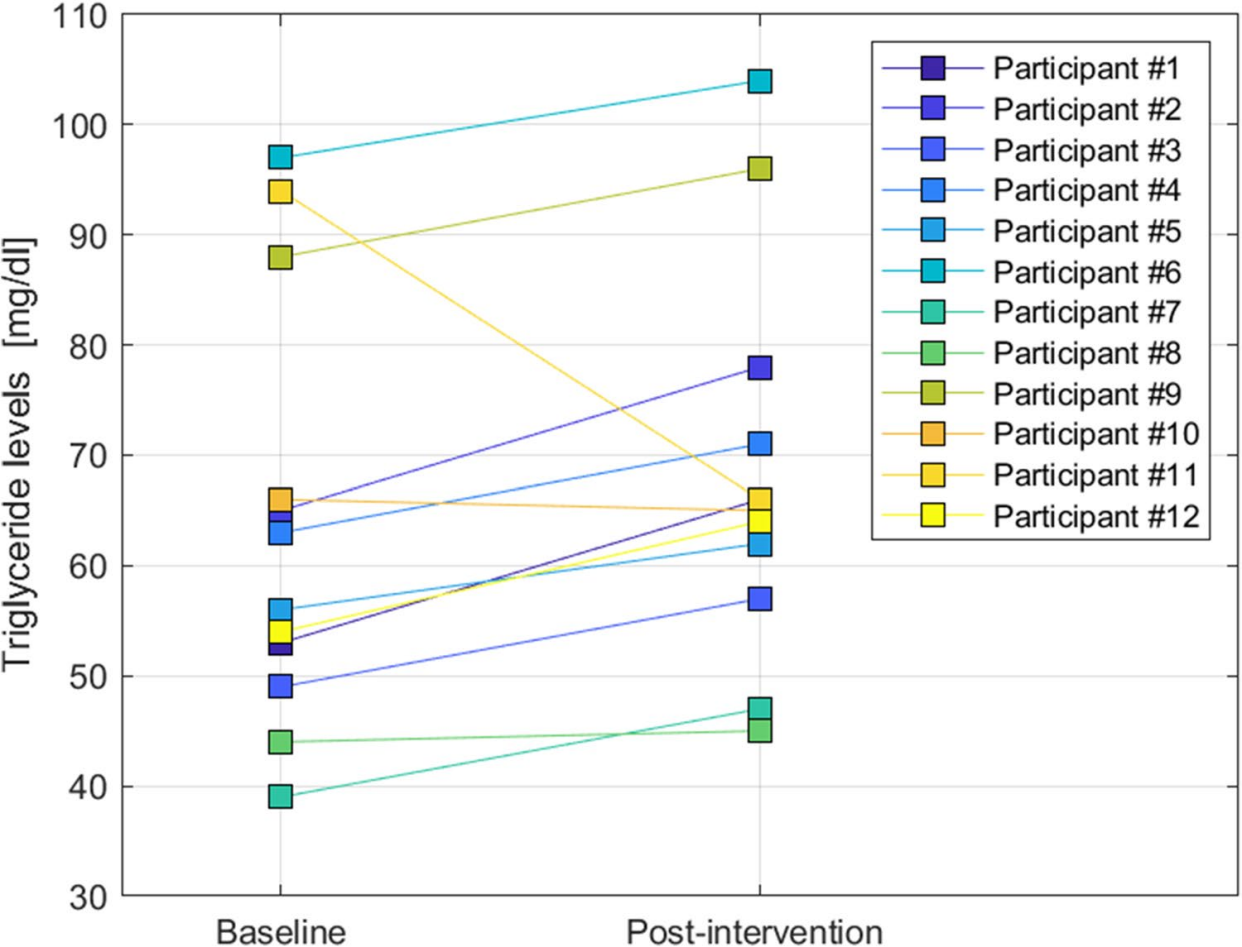
Inter-Individual differences and the extent and direction of individual changes in triglyceride levels before and after treatment. The 12 colors depict the 12 participants of the study where triglyceride levels at baseline (in mg/dl) are shown on the left, and triglyceride levels post-intervention (in mg/dl) are shown on the right. Under continuous 24 h/day monitoring and the continuation of standardized diet, sleep/wake cycle, and physical activity, triglyceride levels increased in 10 of 12 participants and decreased in 2 of 12 individuals

Triglycerides showed a statistically significant increase from 64.0 ± 19.3 mg/dl at baseline to 68.4 ± 17.4 mg/dl after administration of 30 mg mirtazapine for 7 days (mean change + 4.4; 95% CI [– 11.4, 2.6]; *p* = 0.044; *Z* = – 2.0). As depicted in Fig. 2, triglycerides increased in 10 of 12 participants and decreased in 2 of 12 individuals.

HDL-C decreased from 47.5 ± 5.6 mg/dl at baseline to 43.3 ± 5.5 mg/dl at day seven (mean change – 4.3; 95% CI [2.1, 6.5]; *p* = 0.004; *Z* = – 2.9). HDL-C decreased in 11 of 12 participants and increased in 1 of 12 participants. The TG/HDL-C ratio significantly increased from 1.4 ± 0.4 mg/dl to 1.6 ± 0.5 mg/dl (mean change + 0.2; 95% CI [– 0.4, 0.1]; *p* = 0.019; *Z* = – 2.4). TG/HDL-C ratio increased in 11 of 12 participants and decreased in 1 of 12 participants. LDL-C decreased from 85.8 ± 20.8 mg/dl at baseline to 77.2 ± 17.2 mg/dl (mean change – 8.7; 95% CI [3.8, 13.5]; *p* = 0.008; *Z* = – 2.7). LDL-C decreased in 9 of 12 participants, increased in 1 of 12 individuals and did not change in 2 of 12 participants. Non-HDL-C significantly decreased from 99.0 ± 23.5 mg/dl to 91.1 ± 19.4 mg/dl (mean change – 8.0; 95% CI [1.9, 14.0]; *p* = 0.023; *Z* = – 2.3). A decrease in non-HDL-C was observed in 10 of 12 participants, in 2 of 12 non-HDL-C increased. Total cholesterol decreased from 146.6 ± 25.1 mg/dl at baseline to 134.3 ± 21.1 mg/dl at day 7 (mean change – 12.3; 95% CI [5.4, 19.1]; *p* = 0.005; *Z* = – 2.8). Total cholesterol decreased in 10 of 12 individuals, increased in 1 of 12 participants and remained unchanged in 1 of 12 participants.

### Anthropometric measures

Body weight significantly decreased from 74.4 kg to 73.8 kg (mean change – 0.6 kg; 95% CI [0.4, 0.8]; *p* = 0.002; *Z* = – 3.1), and BMI significantly decreased from 22.8 to 22.6 (mean change – 0.2; 95% CI [0.1, 0.2]; *p* = 0.002; *Z* = – 3.1). The decrease in body weight and BMI was very systematic and observed in all 12 participants. Waist circumference (mean change – 0.4 cm; 95% CI [– 2.1, 2.9]; *p* = 0.838; *Z* = -0.2) and waist-to-hip ratio (mean change 0.0; 95% CI [– 0.0, 0.0]; *p* = 0.814; *Z* = – 0.2) as a surrogate marker for central adiposity did not significantly change.

## Discussion

### Main finding

This is the first analysis to report unfavorable changes in biomarkers for atherogenic dyslipidemia in healthy male participants in the context of weight loss following a 7-day administration of 30 mg/d mirtazapine. This observation is remarkable and provides insights into the short-term effects of mirtazapine on lipid metabolism that are independent of weight gain.

While weight gain and metabolic derangement are a well-described side effect of mirtazapine treatment in individuals with psychiatric disorders, (Himmerich et al. 2015) the effect of mirtazapine on lipid metabolism in healthy participants is less investigated. In healthy individuals, there is only one comparable study that investigated changes in lipid metabolism related to mirtazapine treatment. Nicholas and colleagues found that a 4-week treatment with mirtazapine 15 mg per day, which was increased to 30 mg per day at the beginning of week two, resulted in a significant rise in plasma triglycerides and weight gain in 50 healthy individuals using a placebo-controlled study design (Nicholas et al. 2003). Aligning with the observation of Nicholas et al., in our study, we observed a significant rise in triglycerides. However, contrary to their findings, triglycerides increased in our participants in the absence of weight gain. We observed a small but systematic weight loss in all participants. The reasons for the slight weight loss observed under dietary clamping and unchanged energy expenditure remains unclear. The expected effect of weight loss and loss of intrahepatic fat content in particular on the lipid profile would be a decrease in serum triglyceride levels/triglyceride-rich lipoproteins (Chan et al. 2008; Borén et al. 2020). However, despite a slight weight loss, triglycerides significantly increased in our study participants. While none of the healthy study participants met criteria for dyslipidemia after exposure to mirtazapine, these findings are of potential clinical importance for two major reasons. First, the changes became evident after a very short period on mirtazapine, raising concerns about long-term effects on lipid metabolism. This question about long-term effects of mirtazapine on blood lipid levels in the context of stable weight or weight loss cannot be addressed with this study but warrants further research. Second, and importantly, biomarkers of atherogenic dyslipidemia [triglycerides (increased) and HDL-C (decreased)] worsened in our study in the context of weight loss. It appears that effects of short-term exposure to mirtazapine outweigh the lipid changes that would be expected in the context of weight loss (Chan et al. 2008; Borén et al. 2020). Taken together, our findings suggest potential effects of mirtazapine on hepatic lipid metabolism independent of weight gain.

The effects we observed in healthy individuals with short-term exposure to mirtazapine may be more pronounced and of higher clinical relevance in individuals with depressive disorders with an elevated risk for metabolic disorders (Vancampfort et al. 2015; Pan et al. 2012) and/or individuals with pre-existing metabolic conditions or prolonged treatment with mirtazapine. In addition, mirtazapine induced increases in appetite and cravings for sweet foods as reported previously (Hennings et al. 2019) might result in weight gain under normal dietary conditions and add to drug-induced dyslipidemia (Lechner et al. 2020a; Hieronimus and Stanhope 2020). This may overall contribute to elevation of cardiovascular risk in addition to cardiovascular risk already associated with psychiatric conditions such as major depression (Penninx and Lange 2018).

Mechanistically, the effect of mirtazapine on triglyceride levels may in part be explained by disinhibition of insulin secretion by mirtazapine due to inhibition of pancreatic ß-cell α2-adrenoceptors (Hennings et al. 2012). As reported previously, as expected in the context of disinhibition of insulin secretion, we observed increased insulin release with mirtazapine in our study (Hennings et al. 2019). This aligns with the observed triglyceride increase since insulin stimulates hepatic de novo lipogenesis and VLDL-secretion [serum triglycerides can be considered as a surrogate for VLDL in this study (VLDL = triglycerides/5)] (Lechner et al. 2020a; Titchenell et al. 2016; Samuel and Shulman 2016). Furthermore, increased insulin and triglyceride levels and decreased HDL-C might account for our observation of a decrease in LDL-C after mirtazapine exposure potentially explained by this mechanism: A rise in TG/HDL-C ratio, as we observed in our analysis, implies compositional changes of LDL-particles due to a metabolic shift which renders LDL particles cholesterol-depleted and triglyceride-enriched (Lechner et al. 2020a). This results in a decrease in LDL-C (i.e., lower LDL-cholesterol content) (Lechner et al. 2020a). TG/HDL-C ratio is a metabolic index that serves as a surrogate for atherogenic dyslipidemia, which encompasses a constellation of lipoprotein abnormalities including high serum triglycerides, low HDL-C, and an atherogenic lipoprotein phenotype, including a predominance of small, cholesterol-depleted LDL-P, and an accumulation of triglyceride-rich lipoproteins (Lechner et al. 2020a). Higher TG/HDL-C ratio (i.e., high triglycerides and low HDL-C) is associated with both insulin resistance and measures of atherogenic dyslipidemia, which includes a smaller LDL-P diameter, higher remnant lipoprotein particle cholesterol, and further traits that render low density lipoproteins more atherogenic such as increased ApoC3 (Lechner et al. 2020a; Arnold et al. 2021). In patients with coronary artery disease, the TG/HDL-C ratio was significantly higher in patients with thin-cap fibroatheromas than in those without, and it was non-significantly higher in patients with multiple recurrent acute coronary syndromes than in those with long-standing stable angina (Lechner and Halle 2019; Vergallo et al. 2019a, 2019b).

Overall, although our study included healthy individuals neither meeting the criteria for atherogenic dyslipidemia or hypertriglyceridemia, nor carrying any common vascular risk factors as documented by a thorough clinical assessment, we observed significant adverse short-term changes in lipoprotein pattern which may in part be explained by increased drug-induced insulin release as shown in our prior analysis (Hennings et al. 2019). Extrapolating these changes that occurred in a short period in healthy individuals, and considering the effects of mirtazapine on weight gain without caloric restriction, it is likely possible that these effects are clinically significant in the long run, i.e., under longer term antidepressant treatment and/or in the context of pre-existing metabolic conditions (Lechner et al. 2020a; Ganda et al. 2018). These alterations associated with mirtazapine exposure may have the potential to contribute to elevated cardiovascular risk with longer administration of the drug as it is common in treatment of individuals with depressive or anxiety disorders. Of note, in the context of pre-existing metabolic disorders as frequently observed as a comorbidity in psychiatric patients and in the absence of dietary restriction as in this study, (Penninx and Lange 2018) mirtazapine-related changes in lipid metabolism are likely even more pronounced. Additionally, mirtazapine induced appetite, and cravings for sweet foods in particular in our participants (Hennings et al. 2019) that would have exacerbated drug-induced dyslipidemia mediated by weight gain and/or increased intrahepatic fat content under free-running conditions (Lechner et al. 2020a; Hieronimus and Stanhope 2020).

### Limitations and strengths

Our analysis is limited to short-term effects of mirtazapine on lipid metabolism, and additional research investigating long-term effects is warranted since mirtazapine is a medication for longer term use. Given the small sample size of our study, type II error cannot be excluded. Further, our study design does not include a comparator group limiting the interpretation and generalizability of the results. Due to the exploratory nature of our study and limited pre-existing research in this area, a power analysis was not feasible for this descriptive analysis. In addition, it is difficult to interpret the clinical significance of the minimal, though statistically significant changes in lipid metabolism. However, the lifetime exposure model of cardiovascular diseases suggests that exposure to minimal changes in risk factors over a lifetime and/or over a long period in a lifetime and may cumulatively have a large impact on clinically relevant endpoints (Lechner et al. 2020b). Lastly, we do not report intermediate measurements of changes in the lipid profile—therefore, we cannot assess the dynamics and whether the maximum effect of mirtazapine on the lipid profile may possibly have been reached even more short term. Thus, our results should be regarded as explorative and hypothesis-generating and require replication and confirmation in larger studies with longer duration and more assessment points as well as studies including a comparator group.

Strengths of our study include the highly standardized conditions and the use of an “extremely” healthy and homogenous study population. The highly standardized study design minimizes the risk of external confounders such as varying caloric intake, physical activity patterns and circadian rhythms which all impact on body weight and lipid metabolism and thus are potential confounders when investigation the impact on mirtazapine on body weight and lipid metabolism. This analysis may therefore be a first step towards disentangling the direct, weight-independent pharmacological effects of mirtazapine on lipid metabolism.

## Conclusion

In conclusion, this is the first study showing unfavorable changes in lipid metabolism following a 7-day exposure to mirtazapine under highly standardized conditions in healthy participants and in the absence of weight gain. These findings support the hypothesis that alterations in lipid metabolism may be explained by primary pharmacological effects of mirtazapine independent of and in addition to known secondary effects associated with medication-induced weight gain. Due to the clinical relevance of these findings indicating potential contribution to cardiovascular risk, further research is warranted including validation in larger cohorts or investigation of effects on lipid metabolism in individuals with mental health conditions where treatment with antidepressants is common such as depressive or anxiety disorders.

## Clinical perspectives

### Competency in patient care

Our findings support the hypothesis that a direct pharmacological effect of mirtazapine may contribute to alterations in lipid metabolism independent of secondary effects related to medication-induced weight gain. Treatment with mirtazapine should be carefully considered in individuals at increased cardiometabolic risk and metabolic monitoring is advised.

### Translational outlook

Further research including metabolomics approaches may additionally improve our understanding of antidepressants’ effects on lipid metabolism and may eventually inform prediction of metabolic risk, monitoring strategies, and individualized treatment.

## Data Availability

Due to the GDPR regulations and local data protection laws we cannot provide patient data.
